# Diagnostic Accuracy of Apparent Diffusion Coefficient and ^123^I-Metaiodobenzylguanidine for Differentiation of Multiple System Atrophy and Parkinson’s Disease

**DOI:** 10.1371/journal.pone.0061066

**Published:** 2013-04-17

**Authors:** Atsushi Umemura, Tomoko Oeda, Ryutaro Hayashi, Satoshi Tomita, Masayuki Kohsaka, Kenji Yamamoto, Hideyuki Sawada

**Affiliations:** 1 Clinical Research Center, National Hospital of Utano, Kyoto, Japan; 2 Department of Neurology, National Hospital of Utano, Kyoto, Japan; University of Cambridge, United Kingdom

## Abstract

**Background:**

It is often hard to differentiate Parkinson’s disease (PD) and parkinsonian variant of multiple system atrophy (MSA-P), especially in the early stages. Cardiac sympathetic denervation and putaminal rarefaction are specific findings for PD and MSA-P, respectively.

**Purpose:**

We investigated diagnostic accuracy of putaminal apparent diffusion coefficient (ADC) test for MSA-P and ^123^I-metaiodobenzylguanidine (MIBG) scintigram for PD, especially in early-stage patients.

**Methods:**

The referral standard diagnosis of PD and MSA-P were the diagnostic criteria of the United Kingdom Parkinson’s Disease Society Brain Bank Criteria and the second consensus criteria, respectively. Based on the referral standard criteria, diagnostic accuracy [area under the receiver-operator characteristic curve (AUC), sensitivity and specificity] of the ADC and MIBG tests was estimated retrospectively. Diagnostic accuracy of these tests performed within 3 years of symptom onset was also investigated.

**Results:**

ADC and MIBG tests were performed on 138 patients (20 MSA and 118 PD). AUC was 0.95 and 0.83 for the ADC and MIBG tests, respectively. Sensitivity and specificity were 85.0% and 89.0% for MSA-P diagnosis by ADC test and 67.0% and 80.0% for PD diagnosis by MIBG test. When these tests were restricted to patients with disease duration ≤3 years, the sensitivity and specificity were 75.0% and 91.4% for the ADC test (MSA-P diagnosis) and 47.7% and 92.3% for the MIBG test (PD diagnosis).

**Conclusions:**

Both tests were useful in differentiating between PD and MSA-P, even in the early stages. In early-stage patients, elevated putaminal ADC was a diagnostic marker for MSA-P. Despite high specificity of the MIBG test, careful neurological history and examinations were required for PD diagnosis because of possible false-negative results.

## Introduction

Differential diagnosis of Parkinson’s disease (PD) and the parkinsonian variant of multiple system atrophy (MSA-P) is often hard in the early stages of the disease, even for movement disorder specialists [1], [2]. The most distinct brain pathological difference between PD and MSA-P is diffuse rarefaction of the putamen, which reflects severe neuronal loss with astrogliosis and iron accumulation in the neuropil of the putamen, especially in the dorsolateral portion [3]. These pathological changes, rarefaction and iron accumulation, are detected as dorsolateral putaminal hyperintensity in T2-weighted magnetic resonance imaging (MRI) [4]–[6] and low signal change in T2*-weighted MRI [7], respectively, in the advanced stage, but are not detected clearly in the early stage of disease [8]. Brain MRI apparent diffusion coefficient (ADC) reflects hydrogen motility that is elevated in the region of neurodegeneration [9], and even in the early stage of MSA-P, pathological changes developed in the putamen could be detected as elevation of ADC. Previous studies have demonstrated the efficacy of the putaminal ADC values in discrimination of MSA-P from PD [10], [11], even in the early stage [12].

Another radiological diagnostic test to differentiate PD from MSA-P is based on degeneration of peripheral sympathetic nerve terminals [13]–[15]. The uptake of ^123^I-metaiodobenzylguanidine (MIBG), a norepinephrine analog, into the myocardium is reduced in patients with PD [16]–[20], owing to denervation of the cardiac sympathetic postganglionic fibers. MIBG uptake into the myocardium reflects the density of postganglionic sympathetic nerve endings [21]. The degree of cardiac sympathetic denervation in PD patients is positively associated with disease progression [19], [22], [23]. The relative ratio of radioisotope uptake into the heart to that in the mediastinum (H/M ratio) in MIBG scintigraphy is effective for making a differential diagnosis between PD and MSA in the early stage of the disease [15].

Comparing the diagnostic accuracy of the cardiac MIBG test and that of the putaminal ADC test, it is reported that the latter is superior for differential diagnosis between MSA-P and PD [24]; however, this has not been investigated in the early stage of the disease. The purpose of the present study was to investigate the diagnostic accuracy of these tests when applied to early-stage patients. The most discriminative cut-off points for ADC and MIBG scintigraphy in MSA-P and PD patients were determined, and the diagnostic accuracy of the tests was assessed by applying these cut-off points to patients with disease duration ≤3 years.

## Materials and Methods

### Study Design

A retrospective study was conducted to investigate diagnostic accuracy of the putaminal ADC test for MSA-P and cardiac MIBG test for PD. Referral standard diagnosis of PD and MSA-P was made according to the United Kingdom Parkinson’s Disease Society Brain Bank Clinical Diagnostic Criteria (steps 1 and 2) [25] and the second consensus clinical diagnostic criteria [26], respectively. According to these criteria, the referral diagnosis was made carefully by three expert neurologists (TO, ST, and HS) who were masked to the result of ADC and MIBG tests. Collecting all medical records until December 2012 for the referral diagnostic tests, the diagnosis was made when the diagnoses by three experts were consistent, and the diagnosis was undetermined when they were inconsistent. Based on the referral diagnostic criteria, the specificity, sensitivity, positive likelihood ratio, negative likelihood ratio, and the area under the receiver-operator characteristic (ROC) curve (AUC) of the two index tests, the putaminal ADC test and cardiac MIBG test, were obtained and the most discriminating cut-off points were determined using the ROC curves. When applying these cut-off points to test results obtained from early patients with disease duration of ≤3 years, the diagnostic accuracy was calculated to investigate early diagnostic usefulness.

### Study Subjects

We enrolled 260 consecutive patients with muscular rigidity, hand or leg tremor, or slowed movements who had undergone both ADC and MIBG scintigraphy at the Department of Neurology of Utano National Hospital between January 2001 and October 2010. Patients with a history of diabetes mellitus, myocardial infarction, current heart diseases including heart failure and cardiomyopathy, and current use of monoamine oxidase B inhibitors, droxidopa or tricyclic antidepressants were excluded, which can interfere with MIBG imaging [27], and patients with MRI findings of other putaminal lesions (hemorrhages, infarcts, hemangioma or other tumors) were also excluded, which can influence the ADC test [9]. The study was approved by the Bioethics Committee of Utano National Hospital, and written informed consent was obtained from each participant. Patients’ characteristics including age at study enrollment, sex, age of onset, disease duration, disease severity [Hoehn and Yahr (H-Y) stage, and Unified Parkinson’s Disease Rating Scale Part III (UPDRS III) for PD, and Unified Multiple System Atrophy Rating Scale Part II (UMSARS II) for MSA-P], presence of orthostatic hypotension, and levodopa daily dose [28] were collected. The definition of orthostatic hypotension was according to UPDRS IV.

To determine the normal range of the putaminal ADC in our hospital, age-matched controls (patients reporting nonspecific symptoms including headache, or dizziness, but with no evidence of active neurological diseases) were recruited [*n* = 29 (14 male); mean (SD) age, 67.6 (12.6) years]. Concerning the cardiac MIBG test, we referred to our previous study due to the risk of radiation exposure and estimated the mean (SD) H/M ratio to be 2.45 (0.40) in control patients [*n* = 20 (10 male); age, 66.7 (13.5) years] [29].

### Protocol for ADC Acquisition

Axial diffusion-weighted imaging (DWI) scans (1.5 T Magnetom Symphony; Siemens, Erlangen, Germany) were obtained (slice thickness = 5 mm, interslice gap = 0.5 mm) using a single-shot echoplanar imaging (EPI) sequence [repetition time (TR) = 4,200 ms, echo time (TE) = 90 ms, matrix size = 128×128 mm^2^] with diffusion-sensitizing gradients switched in three orthogonal (slice, z; read-out, x; phase-encoding, y) directions and two different b values (0 and 1,000 s/mm^2^). The total DWI scan time was 1 min 11 s. The ADC map was generated by calculating the mean of three orthogonal directions. Regional ADC values were determined by mid-lateral part of the putamen. The region of interest was a fixed circle, and the area was 10.0 mm^2^.

T2-weighted images were obtained at the same time using a turbo spin echo technique (TR = 3,420 ms, TE = 129 ms, matrix size = 196×320 mm^2^, acquisition time = 2 min 35 s) and carefully reviewed to avoid areas of leukoencephalopathy, edema or infarction.

### Protocol for Cardiac Scintigram of MIBG

To investigate cardiac sympathetic nerve terminal denervation, 3 mCi (111 MBq) ^123^I-MIBG was injected intravenously into each patient in the supine position using a gamma camera (Symbia E; Siemens) equipped with low middle energy general purpose collimators. A 15% window was centered on 159 keV for imaging. A planar image of the chest was obtained at 4 h after injection (late image) because it had higher sensitivity and specificity for diagnosis of PD [19], [21]. The regions of interest were located in the heart and the upper mediastinum on a planar image according to plain chest X-ray. Relative cardiac accumulation was calculated as H/M ratio at 4 h.

### Determination of a Cut-off Point of ADC or H/M as a Diagnostic Marker and Diagnostic Accuracy

To evaluate the diagnostic accuracy of each test, we determined the cut-off points for all collected data. Changing cut-off points, sensitivity and specificity were calculated and ROC curves were obtained. According to the ROC curves, we determined the most discriminative cut-off point of each test, and at the cut-off points, the sensitivity, specificity, and positive and negative likelihood ratios were calculated.

To investigate diagnostic accuracy in early-stage patients, the diagnostic parameters were calculated and restricted to the data that were obtained within 3 years of disease onset. The disease duration was defined as the period from the occurrence of initial symptoms and signs of the disease to the date of the tests. When the tests were performed twice or more, the first-time test was incorporated and the other data were excluded.

### Statistical Analysis

Image and data acquisition was performed by an investigator (AU) who was masked to the clinical diagnosis. Owing to its non-Gaussian distribution, the difference in H/M or ADC in the groups of patients with PD and MSA-P was statistically analyzed using the nonparametric Mann–Whitney *U* test. The correlation between H/M or ADC and disease duration and severity was analyzed using a general linear model after adjustment for age at study enrollment and sex. Age at examination and disease duration were compared using the nonparametric Mann–Whitney *U* test. All statistical analyses were carried out using GraphPad Prism for Windows ver. 5.0 (GraphPad Software, San Diego, CA, USA, http://www.graphpad.com) and the statistical software program SPSS 18.0 (PASW statistics, http://www.spss.com/). Data were expressed as mean ± SD. A value of *P*<0.05 was considered statistically significant.

## Results

### Characteristics of Subjects

**Figure 1:**
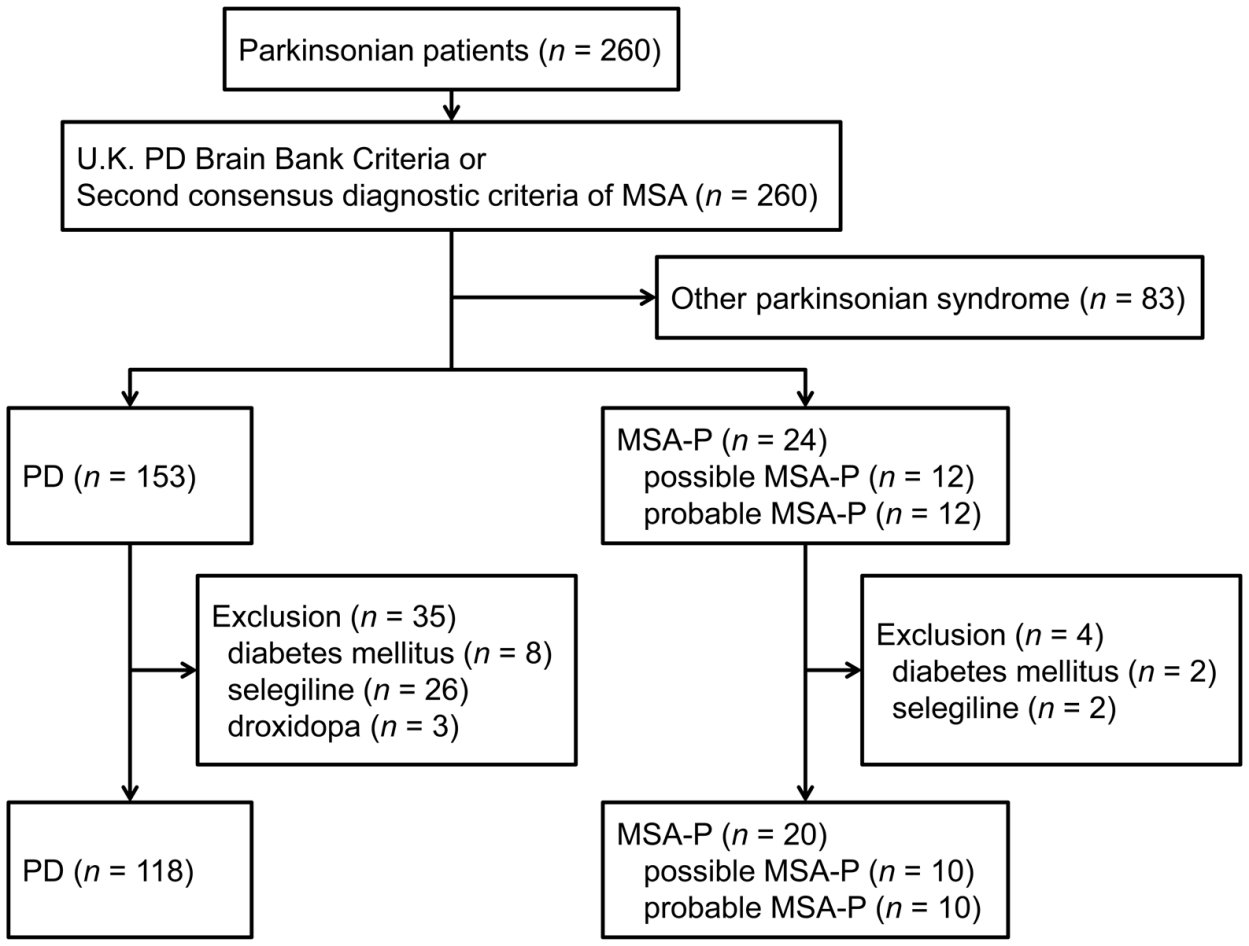
Flow diagram of the eligible patients and the enrollment process of the study. Eligible patients were 260 consecutive patients who underwent both ADC test and MIBG scintigraphy because of extrapyramidal signs. According to the UK Brain Bank criteria of Parkinson’s Disease and the second consensus statement on the diagnosis of MSA as a reference standard, 153 patients had PD, 24 had MSA-P, and the remaining 83 patients had another condition. Of the 153 patients with PD, 35 with a history of diabetes mellitus (*n* = 8) and current use of selegiline (*n* = 26) or droxidopa (*n* = 3) were excluded. Four patients with MSA-P were excluded (two with diabetes mellitus and two for use of selegiline). ADC and MIBG tests were performed in a total of 138 patients.

Among the 260 enrolled participants, 153 were diagnosed with PD and 24 with MSA-P (12 as possible and 12 as probable), according to the referral standard diagnostic criteria, and 83 were diagnosed with another condition (36 progressive supranuclear palsy, 12 dementia with Lewy bodies, nine corticobasal degeneration, 10 cerebellar variant of MSA and 16 unclassified parkinsonism). Of the 153 patients with PD, 35 were excluded owing to history of diabetes mellitus (*n* = 8) and current use of selegiline (*n* = 26) or droxidopa (*n* = 3). Four patients with MSA-P were also excluded (two for diabetes mellitus and two for use of selegiline) (Figure 1). Demographic and clinical data of patients are given in Table 1 and Tables S1 and S2. There was no significant difference in age at examination (*P* = 0.813 for ADC test and *P* = 0.735 for MIBG test). Disease duration was significantly longer in the PD group (*P* = 0.005 for ADC test and *P* = 0.014 for MIBG test). Of the 118 patients with PD, 98 for ADC test and 97 for MIBG test were akinetic-rigid type and 35 for ADC test and 31 for MIBG test had symptomatic orthostatic hypotension. A total of 20 patients with MSA-P were akinetic-rigid type and nine had orthostatic hypotension.

**Table 1:** Demographic and clinical data of the study participants.

	PD (n = 118)	MSA-P (n = 20)	*P*
**Male, n [%]**	47 [39.8]	8 [40.0]	1
**Age of onset, Y [SD]**	60.8 [9.9]	64.6 [8.2]	0.104
**ADC test**			
**Age at ADC test, Y [SD]**	67.5 [9.3]	68.2 [8.2]	0.813
**Disease duration, Y [SD]**	6.8 [4.9]	3.6 [1.8]	0.005
**Disease severity, n [%]**	H–Y **≤**3, 103 [87.3]	Physically independent, 11 [55.0]	NA
	H–Y ≥4, 15 [12.7]	Aid-requiring, 9 [45.0]	
**Motor examination, [SD]**	UPDRS III, 22.4 [9.9]	UMSARS II, 29.7 [12.8]	NA
**Phenotype, akinetic-rigid n [%]**	98 [83.1]	20 [100]	NA
**L-DOPA equivalent dose, mg [range]**	426 [0–1261]	500 [0–900]	NA
**L-DOPA dose, mg [range]**	290 [0–800]	473 [0–900]	NA
**Orthostatic hypotension, n [%]**	35 [29.7]	9 [45.0]	0.199
**MIBG test**			
**Age at MIBG test, Y [SD]**	67.0 [9.2]	67.8 [8.2]	0.735
**Disease duration, Y [SD]**	6.1 [4.7]	3.3 [1.6]	0.014
**Disease severity, n [%]**	H–Y **≤**3, 104 [88.1]	Physically independent, 12 [60.0]	NA
	H–Y ≥4, 14 [11.9]	Aid-requiring, 8 [40.0]	
**Motor examination, [SD]**	UPDRS III, 22.1 [9.5]	UMSARS II, 29.7 [12.8]	NA
**Phenotype, akinetic-rigid n [%]**	97 [82.2]	20 [100]	NA
**L-DOPA equivalent dose, mg [range]**	387 [0–1050]	475 [0–900]	NA
**L-DOPA dose, mg [range]**	263 [0–800]	448 [0–900]	NA
**Orthostatic hypotension, n [%]**	31 [26.3]	9 [45.0]	0.111

Among 138 ADC tests, 47 were performed within 3 years of disease onset, 12 in patients with MSA-P, and 35 with PD. Data from 57 MIBG tests were acquired within 3 years of disease onset, 13 tests in MSA-P and 44 in PD patients.

### Diagnostic Test for MSA-P by ADC of the Putamen

**Figure 2:**
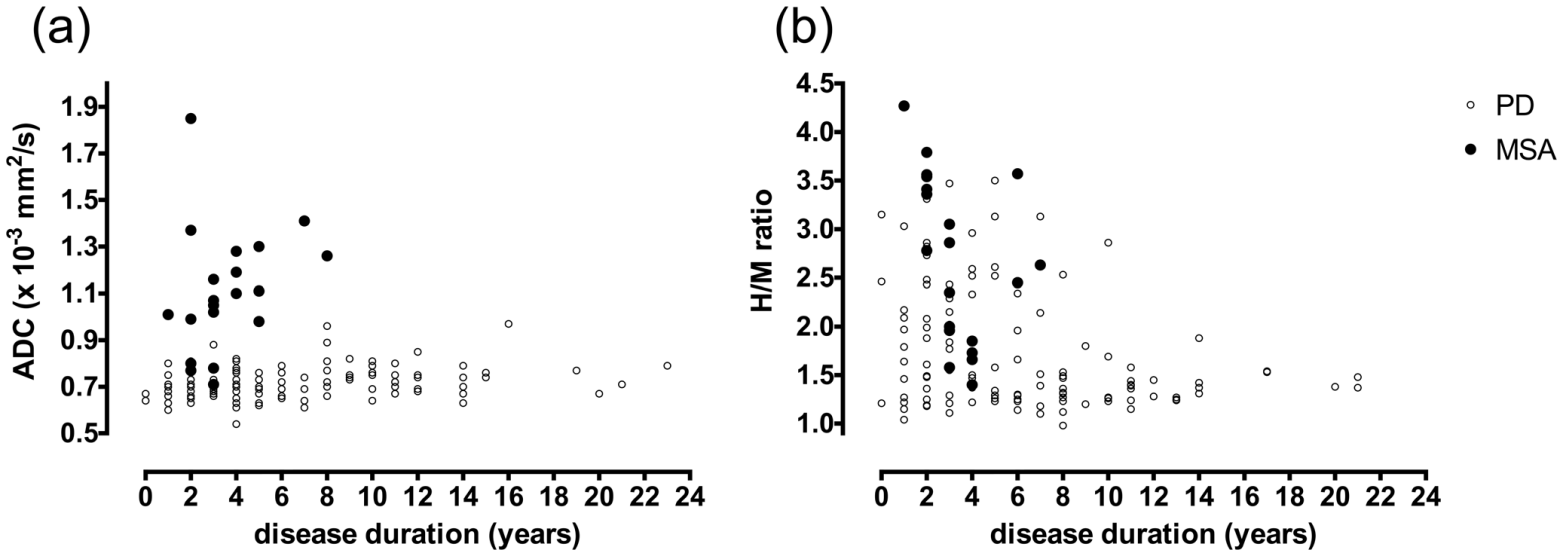
Relationship of ADC and MIBG tests to disease duration. Relationship of the ADC (a) and MIBG (b) tests to duration of PD (open circles) and MSA-P (filled circles). The putaminal diffusivity in MSA-P increased even in the early stage (a). The H/M ratio in PD decreased with disease duration (b).

The mean (SD) putaminal ADC was 0.72 (0.07) and 1.11 (0.27) in patients with PD and MSA-P, respectively; the difference was highly significant (Table 2, *P*<0.001). The mean (SD) putaminal diffusivity was 0.72 (0.05) in the controls. There was no significant correlation between ADC and disease duration or severity in MSA-P (*P* = 0.242 for disease duration, *P* = 0.360 for severity) (Table S3, Figure 2a).

**Table 2:** Comparison of the putaminal diffusivity (×10^–3^ mm^2^/s) between PD and MSA-P groups.

	Normal range, mean [SD]	PD		MSA-P		*P*
		*n*	mean [SD]	*n*	mean [SD]	
**Total**	0.72 [0.05]	118	0.72 [0.07]	20	1.11 [0.27]	<0.001
**Duration ≤3 years**	NA	35	0.70 [0.06]	12	1.05 [0.31]	<0.001

**Figure 3:**
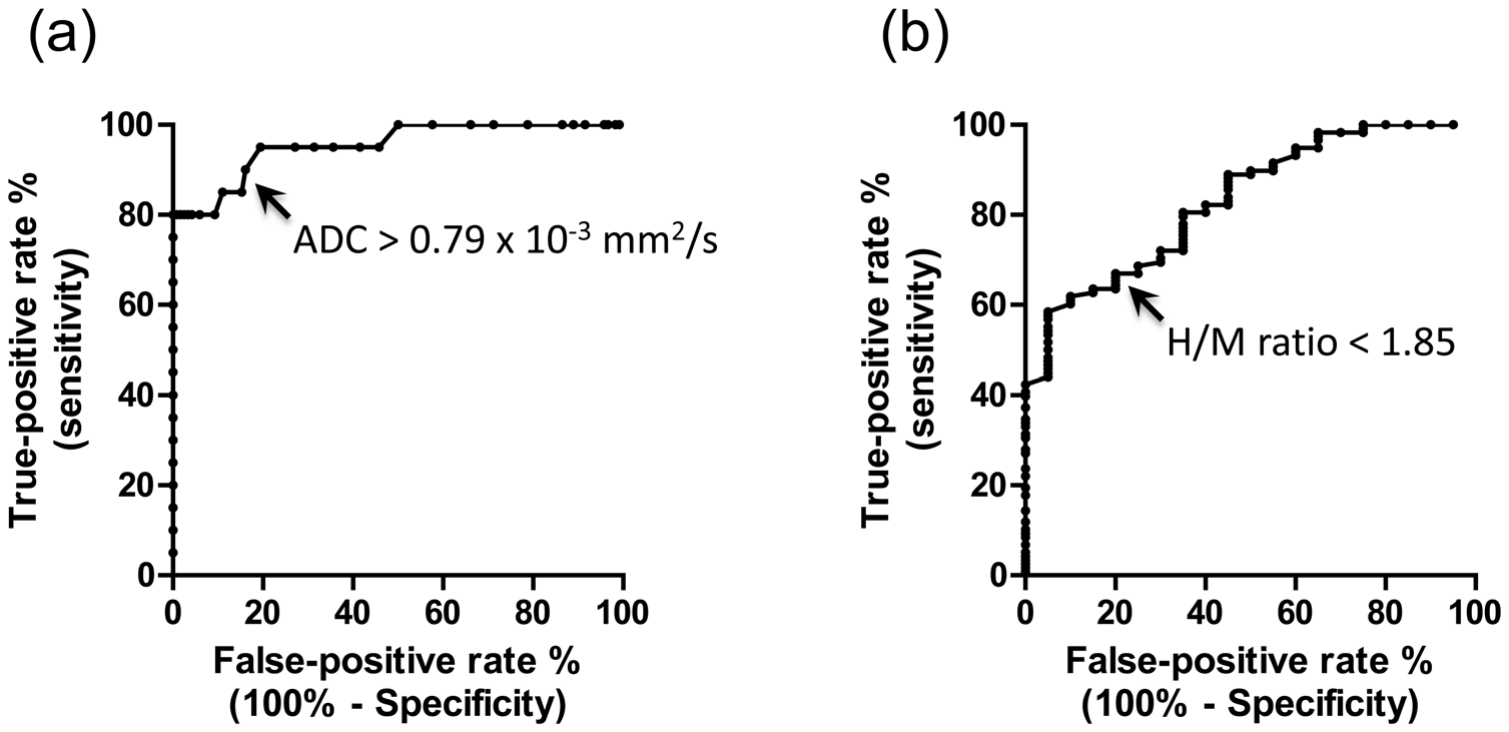
Differentiation of MSA-P and PD. ROC curves of the diagnosing MSA-P by the ADC test (a) and the diagnosing PD by the MIBG test (b). The most discriminative cut-off points (near the left upper corner of the graph) were marked with arrows. The sensitivity and specificity were determined according to the ROC curves.

The AUC was 0.95 [95% confidence interval (CI): 0.90–1.01] for the ADC test. According to the ROC curves, the most discriminative cut-off point for diagnosing MSA-P was 0.79. The sensitivity and specificity was 85.0% (95% CI: 62.1–96.8%) and 89.0% (95% CI: 81.9–94.0%), respectively. The positive and negative likelihood ratios were 7.72 (95% CI: 4.53–13.15) and 0.17 (95% CI: 0.06–0.44) (Figure 3a, Table 3).

**Table 3:** Diagnostic accuracy of the putaminal diffusivity or MIBG in differential diagnosis of MSA-P from PD.

		Putaminal diffusivity	MIBG H/M ratio
	AUC of ROC	0.95	0.83
**Total**	Sensitivity (%)	85	67
	Specificity (%)	89	80
	Positive likelihood ratio	7.72	3.35
	Negative likelihood ratio	0.17	0.41
**Duration ≤3 years**	Sensitivity (%)	75	47.7
	Specificity (%)	91.4	92.3
	Positive likelihood ratio	8.75	6.2
	Negative likelihood ratio	0.27	0.57

### Early Diagnostic Accuracy of MSA by ADC Test

Analyzing the data obtained from MRI that was performed within 3 years of disease onset, the putaminal diffusivity was significantly elevated in patients with MSA-P compared with PD (Table 2). The mean (SD) ADC was 0.70 (0.06) in patients with PD and 1.05 (0.31) in patients with MSA-P; the difference was highly significant (*P*<0.001). With the cut-off point for MSA-P of 0.79, which was determined as shown in Figure 3a, the sensitivity and specificity were 75.0% (95% CI: 42.8–94.5%) and 91.4% (95% CI: 76.9–98.2%), respectively. The positive and negative likelihood ratios were 8.75 (95% CI: 3.09–24.81) and 0.27 (95% CI: 0.11–0.67) (Figure S1a, Table 3).

### Diagnostic Test of PD by MIBG Scintigraphy

Cardiac accumulation of MIBG was significantly reduced in patients with PD compared with MSA-P (Table 4). The mean (SD) cardiac MIBG H/M ratio was 1.75 (0.63) in patients with PD and 2.69 (0.85) in those with MSA-P, and there was a significant difference (*P*<0.001). H/M reduction was significantly correlated with disease duration but not severity in PD (partial η^2^ = 0.104, *P* = <0.001 for disease duration, *P* = 0.233 for severity) (Table S4, Figure 2b).

**Table 4:** Comparison of the MIBG H/M ratio between PD and MSA-P groups.

	Normal range, mean [SD]	PD		MSA-P		*P*
		*n*	mean [SD]	*n*	mean [SD]	
**Total**	2.45 [0.40]	118	1.75 [0.63]	20	2.69 [0.85]	<0.001
**Duration ≤3 years**	NA	44	1.96 [0.66]	13	2.96 [0.80]	<0.001

The AUC was 0.83 (95% CI: 0.75–0.92) for the MIBG test. According to the ROC curves, the most discriminative cut-off point for diagnosing PD was 1.85. The sensitivity and specificity were 67.0% (95% CI: 57.7–75.3%) and 80.0% (95% CI: 56.3–94.3%), respectively. The positive and negative likelihood ratios were 3.35 (95% CI: 1.47–7.65) and 0.41 (95% CI: 0.30–0.58) (Figure 3b, Table 3).

### Early Diagnostic Accuracy for PD by MIBG Test

Cardiac accumulation of MIBG was significantly reduced in patients with PD compared with MSA-P (Table 4). The mean (SD) H/M value was 1.96 (0.66) in patients with PD and 2.96 (0.80) in patients with MSA-P; this difference was significant (*P*<0.001). With the cut-off point of 1.85, the sensitivity and specificity were 47.7% (95% CI: 32.5–63.3%) and 92.3% (95% CI: 64.0–99.8%) and the positive likelihood ratio and negative likelihood ratio were 6.20 (95% CI: 1.33–28.92) and 0.57 (95% CI: 0.41–0.78) (Figure S1b, Table 3).

## Discussion

The putaminal ADC test is based on the diffuse rarefaction of the putamen, and consistent with previous studies, the putaminal diffusivity was significantly elevated in patients with MSA-P (Table 2). The cardiac MIBG test is based on peripheral sympathetic nerve terminals, and cardiac accumulation of MIBG was significantly reduced in patients with PD (Table 4). Although there is the possibility that calcium channel blockers and sympathomimetics as methylphenidate interfere with the uptake of MIBG [27], in the analysis of subgroup excluding patients with these drugs, similar results were obtained and they affected minimal in the present study (data not shown).

Analysis of all the data revealed that the putaminal ADC test was superior to MIBG scintigraphy in sensitivity, whereas the MIBG test was a more specific diagnostic test. In addition, we compared the diagnostic accuracy (sensitivity, specificity, AUC, positive likelihood ratio and negative likelihood ratio) between ADC and MIBG in patients with short disease duration. When applying the test to patients with a disease duration ≤3 years, as well as to all the patients, the putaminal diffusivity of MSA-P was significantly elevated (Table 2) and H/M of PD was significantly reduced (Table 4). The ADC test had high sensitivity and specificity for MSA-P diagnosis, and the accuracy was confirmed in early diagnosis. As well as the ADC test, the MIBG test had high sensitivity and specificity. In early diagnosis of PD, MIBG was highly specific but the sensitivity was low. The most discriminative cut-off point was 1.85 for H/M; the value of which was high when compared with our previous study [19]. We enrolled only patients with PD or MSA-P. The difference in sensitivity for H/M may reflect these cohort variances. Previous pathological studies [30], [31] have shown that some patients with MSA have a slight decrease in cardiac uptake of MIBG; possibly because of mild degeneration of the cardiac sympathetic nerve, and depletion of cardiac sympathetic nerve is closely related to the presence of Lewy bodies in the sympathetic ganglia. In the present study, H/M was decreased mildly in some cases with MSA-P (Figure 2b).

As shown in Figure 2a and Table S3, ADC increased in most patients with MSA-P, even in the early stage, suggesting that putaminal rarefaction has occurred at onset of symptom. In previous studies [32], [33], neurologically normal elderly subjects infrequently (0.4–0.8%) had abundant glial cytoplasmic inclusions consistent with MSA. In this context, putaminal diffusivity may be elevated in the preclinical stage of MSA-P. In contrast, cardiac MIBG accumulation was thought to be markedly reduced at the onset of PD in some cases (Figure 2b), consistent with previous studies demonstrating that PD affects preclinical cardiac sympathetic denervation [21], [34]. These findings suggest that the putaminal diffusivity of MSA-P reflects pathological involvement at disease onset in most cases, and therefore, the onset of cardiac sympathetic denervation seems to be highly variable in PD, which could precede nigrostriatal nerve denervation, as well as following the latter change by >3 years. These suggestions were considered to reflect the difference in diagnostic accuracy between the ADC and MIBG tests, especially in patients with early-stage disease. To avoid false-negative results of the MIBG test, careful neurological examinations are required to make a diagnosis of PD, especially in the early stages.

Although, in the absence of postmortem verification, we cannot exclude possible inconsistency between clinical and pathological diagnoses in some cases, clinical diagnoses were based on stringent diagnostic criteria, and made when the diagnoses by three experts were consistent, thus, making the clinical diagnosis of the patients reliable enough to evaluate diagnostic accuracy.

In summary, our study provides further evidence of the utility of putaminal diffusivity in early diagnosis of MSA-P. The present data indicate its superiority to MIBG scintigraphy.

## Supporting Information

Figure S1 ROC curves in patients with a duration of ≤3 years (a, b). The sensitivity and specificity were investigated according to the set cut-off points from the entire disease cohort (marked with arrows).
(TIF)

Table S1 Demographic and clinical data of the study participants with short disease duration undergoing ADC test.
(XLSX)

Table S2 Demographic and clinical data of the study participants with short disease duration undergoing MIBG test.
(XLSX)

Table S3 Correlation between putaminal diffusivity and clinical factors (disease duration, severity, age, and sex) in patient with MSA-P.
(XLSX)

Table S4 Correlation between MIBG H/M ratio and clinical factors (disease duration, severity, age and sex) in patient with PD.
(XLSX)
