# Supplementary figures and images for: Diagnostic Accuracy of Apparent Diffusion Coefficient and 123I-Metaiodobenzylguanidine for Differentiation of Multiple System Atrophy and Parkinson’s Disease
Source: PLoS One. 2013 Apr 17;8(4):e61066. doi: 10.1371/journal.pone.0061066 (PMC3629185; doi:10.1371/journal.pone.0061066)

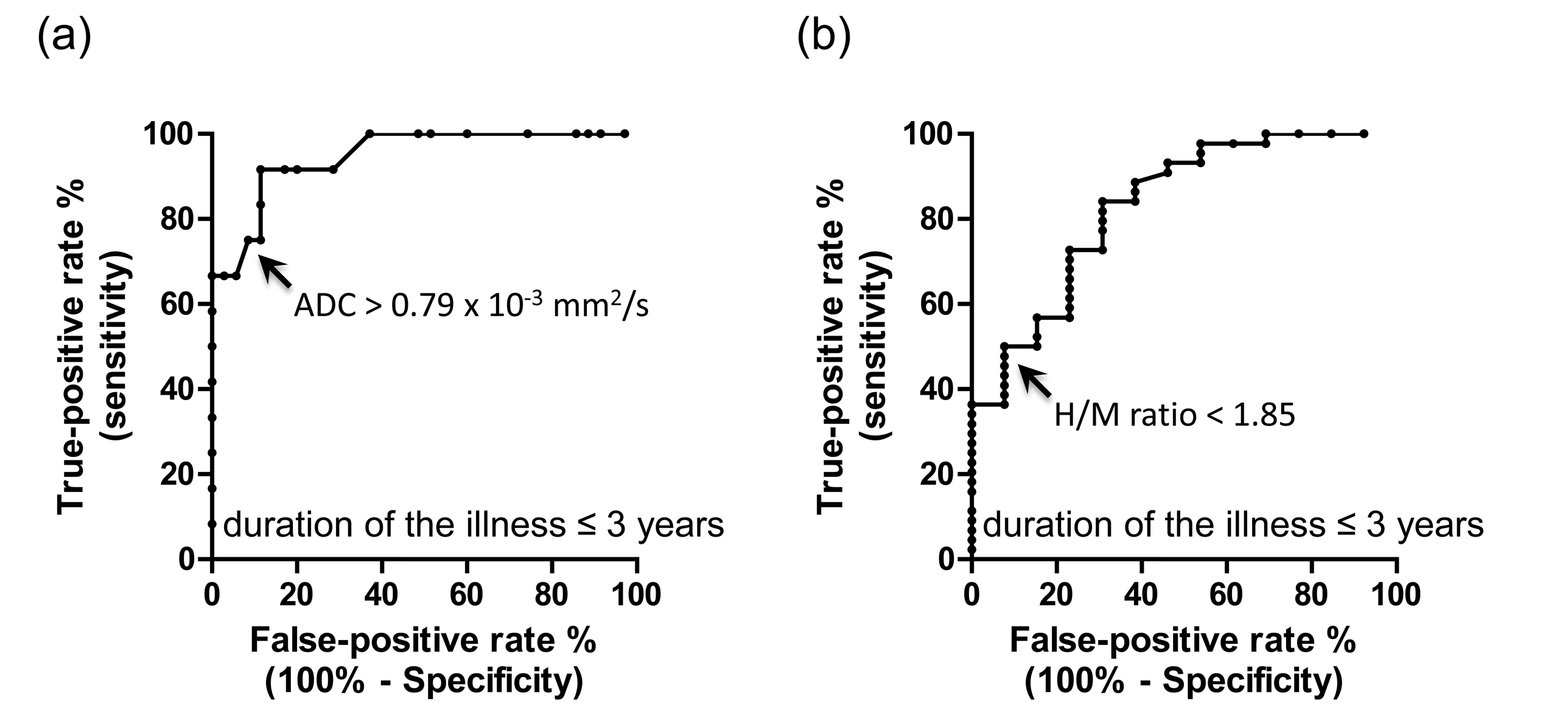

Supplement: Figure S1 — ROC curves in patients with a duration of ≤3 years (a, b). The sensitivity and specificity were investigated according to the set cut-off points from the entire disease cohort (marked with arrows). (TIF) [file pone.0061066.s001.tif]
